# Functional Analyse of GLUT1 and GLUT12 in Glucose Uptake in Goat Mammary Gland Epithelial Cells

**DOI:** 10.1371/journal.pone.0065013

**Published:** 2013-05-28

**Authors:** Qinghua Yu, Liqi Zhu, Jian Lin, Qiang Zhang, Qi Tian, Weiwei Hu, Qian Yang

**Affiliations:** Veterinary College, Nanjing Agricultural University, Nanjing, Jiangsu, PR China; Northwestern University Feinberg School of Medicine, United States of America

## Abstract

Glucose transport, mediated by glucose transporters, is necessary for mammary gland development and lactation. GLUT1 and GLUT12 could both be expressed in the pregnant and lactating mammary gland to participate in the glucose uptake process. In this study, the goat GLUT1 and GLUT12 genes were cloned from Saanen dairy goats and transfected into goat mammary gland epithelial cells to assess their biological functions and distributions. The results showed that both goat GLUT1 and GLUT12 had 12 predicted membrane-spanning helices. Goat GLUT1 and GLUT12 each influenced the mRNA expression of the other transporter and increased the glucose consumption and lactose yield in GLUT1- and GLUT12-transfected goat mammary gland epithelial cells, respectively. The overexpression of GLUT1 or GLUT12 also increased the expression of amino acid transporters SLC1A5, SLC3A2 and SLC7A5 and affected genes expressions in GMGE cells. Using immunofluorescence staining, GLUT1 was detected throughout the cytoplasm and localized to the Golgi apparatus around the nuclear membrane, whereas GLUT12 was mainly distributed in the perinuclear region and cytoplasm. This study contributes to the understanding of how GLUT1 and GLUT12 cooperate in the incorporation of nutrient uptake into mammary gland epithelial cells and the promotion of milk synthesis in the goat mammary gland during lactation.

## Introduction

Glucose is the primary precursor of lactose, the major solid component and osmolarity regulator in milk. Glucose uptake in the mammary glands increases dramatically to meet the requirement for milk synthesis [1], and the increased glucose demand in the mammary gland for lactation is accomplished by increases in the expression of glucose transporters (GLUTs). Facilitative glucose transporters mediate the bidirectional and energy-independent process of glucose transport [2]. Each GLUT has a different transport efficiency, substrate affinity and tissue distribution, indicating that GLUTs have different biological functions in different tissues. However, no data regarding the GLUTs of goat, an important milk-producing animal, are currently available. In lactating goats, up to 85% of the total available glucose is used by the mammary gland [3], which implies that the glucose supply may be the limiting step in lactation.

GLUT1 and GLUT12 are two facilitative glucose transporters that are expressed in the mammary glands [1], [4], and the two transporters cooperate to transport glucose and other hexoses [4]. GLUT1 has a ubiquitous distribution in tissues and culture cells [5]–[7] and is considered to be the primary transporter responsible for basal glucose uptake. GLUT1 expression is detected abundantly in the mammary gland [8] and increases during pregnancy and lactation. However, most of the studies of GLUT1 have focused on tumor cells or established cells, which do not reflect real phenomena in somatic cells [9]–[13]. GLUT12 was originally cloned from the human breast cancer cell line MCF-7 [14] and is localized intracellularly in the bovine mammary epithelial cell line MAC-T [15]. GLUT12 facilitates the transport of glucose with an apparent preferential substrate affinity for glucose over other hexoses [16]. However, in contrast to some other mammals, there is little information regarding the GLUTs in goats.

An understanding of the mechanism and regulation of glucose uptake in the mammary gland is necessary to increase milk production in livestock. In this study, we cloned the goat GLUT1 and GLUT12 genes from goat mammary gland tissue and analyzed the structure of goat GLUT1 and GLUT12 at the genomic and amino acid levels. We also examined whether the cloned goat GLUT1 and GLUT12 cooperate to transport glucose and affect the synthesis of lactose in goat mammary gland epithelial (GMGE) cells.

## Materials and Methods

### Ethics Statement

This study was approved by the Ethical Committee of Animal Experiments of the College of Veterinary Medicine, Nanjing Agricultural University. All animal care and use were conducted in strict accordance with the Animal Research Committee guidelines of the College of Veterinary Medicine, Nanjing Agricultural University. The goats, sacrificed by intravenous injection of sodium pentobarbital euthanasia solution, were obtained from the Shanghai Transgenic Research Center.

### Cloning of goat GLUT1 and GLUT12 and construction of expression vectors

**Figure 1:**
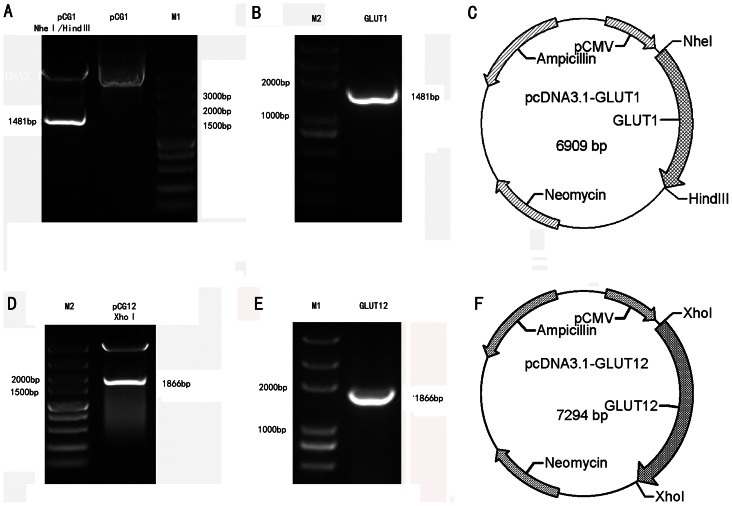
Identification and construction of pcDNA3.1-GLUT1 and pcDNA3.1-GLUT12. (A) Identification of pcDNA3.1-GLUT1 by digestion with *Nhe* I and *Hind* III. M1: DL5000 DNA Marker, pCG1: inserted into pcDNA3.1, pCG1 *Nhe* I + *Hind* III: digested with *Nhe* I and *Hind* III. (B) PCR analysis using primers GLUT1-F/GLUT1-R. M2: Trans2KTM Plus DNA Marker, GLUT1: 1481bp PCR production of goat GLUT1. (C) pcDNA3.1-GLUT1 vector construction diagram. (D) PCR analysis using primers GLUT12-F2/GLUT12-R2. M1: DNA Marker, GLUT12: 1866bp PCR production of goat GLUT12. (E) Identification of pcDNA3.1-GLUT12 by digestion with *Xho* I. M2: Trans2K plus DNA Marker, pCG12 *Xho* I: digested with *Xho* I. (F) pcDNA3.1-GLUT12 vector construction diagram.

Mammary gland tissues were obtained from Saanen dairy goats (*Capra hircus*), and the total RNA was isolated from the mammary gland using the GeneJET™ RNA Purification Kit (Fermentas US CA). The total RNA was treated with RNase-free DNaseI (Fermentas) and used to synthesize the first-strand cDNA. The sequences of all of the primer oligonucleotides used in this study are listed in Table 1. Partial goat GLUT1 and GLUT12 genes were amplified from the first-strand cDNA and cloned into the pJET1.2™ vector (Fermentas) for sequencing. The cDNA was amplified using Phusion™ Hot Start High-Fidelity DNA Polymerase (Fermentas) with the primers GLUT1-F, GLUT1-R, GLUT12-F and GLUT12-R, which were designed based on the 5'- and 3'-untranslated regions (UTRs) of bovine, human and pig GLUT1 or GLUT12. The CDS regions of GLUT1 (1481 bp) and GLUT12 (1866 bp) were amplified from the partial goat GLUT1 and GLUT12 using GLUT1-F1, GLUT1-R1, GLUT12-F1 and GLUT12-R1 and subcloned into the pcDNA3.1 (+) vector to construct pcDNA3.1-GLUT1 and pcDNA3.1-GLUT12 (Fig. 1).

**Table 1:** Sequences of oligonucleotide primers used for PCR and RT-qPCR.

Primers names	Type	Sequences
GLUT1-F	Forward	5'-GCTAGCATGGAGCCCACCAGCAAG -3'
GLUT1-R	Reverse	5'-AAGCTTTCACACTTGGGAATCAGCTCC -3'
GLUT1-F2	Forward	5'-CTGGTTCTGTTCTTCATCTTC-3'
GLUT1-R2	Reverse	5'-CTCCTCAGGTGTCTTGTC-3'
GLUT12-F	Forward	5'-GGAAAAGTGACCGCTCGTG-3'
GLUT12-R	Reverse	5'-TGTCCTGGTAGGCAAAGAACTG-3'
GLUT12-F1	Forward	5'-CTCGAGATGGTACCTGTTGAAAACGCAG -3'
GLUT12-R1	Reverse	5'-CTCGAGTTAGATCTCTGAAGAAAGCTGC -3'
GLUT1-F2	Forward	5'-CTGGTTCTGTTCTTCATCTTC-3'
GLUT1-R2	Reverse	5'-CTCCTCAGGTGTCTTGTC-3'
GLUT12-F2	Forward	5'-TACTCTCCTGTCGTCTGT-3'
GLUT12-R2	Reverse	5'-ATGATGGTGGCTCTTCTC-3'
SWEET1-F	Forward	5'-GTGCTCCTTCAGACTACA-3'
SWEET1-R	Reverse	5'-GTGGTGAGAGGTACATACT-3'
SLC1A5-F	Forward	5'-CCAAGGGACTCTCAAACATTTCA-3'
SLC1A5-R	Reverse	5'-CGGAGGCTAGAACTTTCAAGAA-3'
SLC7A5-F	Forward	5'-ATCGCCGTCTCCTTCTGG-3'
SLC7A5-R	Reverse	5'-CTGGTTCTGGTCGCTTACG-3'
SLC3A2-F	Forward	5'-GCTGCTGCTGCTCTTCTG-3'
SLC3A2-R	Reverse	5'-TGCCACCATCTCTGCTCTG-3'
*ß*-actin-F	Forward	5'-CCAACCGTGAGAAGATGA-3'
*ß*-actin-R	Reverse	5'-CAGAGTCCATGACAATGC-3'

### DNA sequence analysis of goat GLUT1 and GLUT12

The analysis of the cDNA sequences was conducted using the computer program DNASTAR (DNASTAR, Madison, WI), the National Center for Biotechnology Information BLAST site (http://www.ncbi.nlm.nih.gov/BLAST/) and the TMHMM Server v.2.0 program (http://www.cbs.dtu.dk/services/TMHMM/). The multiple sequence alignment was generated using CLUSTALW 2.1.

### GMGE cell isolation and culture

GMGE cells were isolated from the mammary gland tissue of Saanen dairy goats and cultured with the basal growth medium DMEM/F12 containing 10% fetal bovine serum (FBS). Insulin-transferrin-selenium (ITS, 1%) (Invitrogen), 5 mg/mL progesterone (Prospec, ISR, CA), 10^−7^ mol/L hydrocortisone (R&D, CA, USA), 10 ng/mL ovine epithelial growth factor (Prospec) and 5 mg/mL bovine estradiol (Sigma-Aldrich, CA, USA) were added to the basal growth medium to promote the synthesis of milk protein and fat. The mammary epithelial cells were cultured according to the method by Han Hu *et al.* [17].

### Transfection of pcDNA3.1-GLUT1 and pcDNA3.1-GLUT12 into GMGE cells

A 24 µg sample of pcDNA3.1-GLUT1 or pcDNA3.1-GLUT12 was dissolved in 15 mL DMEM/F12 and transfected into GMGE cells using Lipofectamine 2000 (Invitrogen). At 6 h post-transfection, the medium was removed, and the cells were cultured for 2 weeks with GMGE cell culture medium containing G418 (700 µg/ml) until the stably transfected GMGE cells were selected. The GLUT1- and GLUT12-transfected stable GMGE cell lines (GT1-GMGE and GT12-GMGE) were maintained in culture by continuous subculturing.

### Detection of GLUT1, GLUT12, sweet1 and amino acid transporters (AATs) gene expression by RT-qPCR

Total RNA from cells stably transfected with pcDNA3.1-GLUT1, pcDNA3.1-GLUT12 and pcDNA3.1 was obtained, and the 18S and 28S rRNA bands were evaluated following agarose gel electrophoresis. The GeneJET™ RNA Purification Kit (Fermentas) was used with oligo dT and 6-random primers to reverse-transcribe 5 µg of total RNA into cDNA. To determine the gene expression profiles, 2 µL of the cDNA was amplified in a 20 µL reaction mixture containing 10 µL SYBR Premix™ Ex Taq (TaKaRa China CA), 0.4 µL ROX dye II and specific primers (0.4 µM each of the forward and reverse gene-specific primer; Table. 1) using an ABI 7500 instrument. The data are reported as values normalized to the housekeeping gene (β-actin) to account for repeated measures. The differences between the means at each stage of development were determined by Fisher's protected LSD test.

### Detection of glucose absorption and lactose synthesis

The GT1-GMGE, GT12-GMGE and GMGE cells were passaged at the same density in 6-well plates, and the cell culture medium and the total protein content of the cells were sampled at 24 h and 48 h. The glucose and lactose levels in the culture media were determined using the Glucose Assay Kit II and Lactose Assay Kit (Biovision, CA, USA), respectively. The absorbance was measured using a spectrophotometer at a wavelength of 450 or 570 nm. The amount of absorbed glucose or lactose, as determined based on the difference before and after incubation, was expressed as µg of glucose/µg protein or ng of lactose/µg protein.

### Detection of GLUT1 and GLUT12 in GMGE cells

The GT1-GMGE, GT12-GMGE and GMGE cells were grown on coverslips, fixed with 4% paraformaldehyde for 30 min and incubated with 0.1% Triton X-100 for 15 min. Nonspecific reactivity was blocked with 5% BSA (Solarbio China CA) for 1 h at room temperature, and the plates were incubated overnight with mouse monoclonal anti-GLUT1 (1∶50; Abcam, CA, USA) or anti-GLUT12 (1∶50; Santa Cruz, USA) at 4 °C. The coverslips were then washed thoroughly and incubated with Alexa Fluor 488 goat anti-mouse IgG (Invitrogen) or Cy3-labeled IgG (Invitrogen) at 1∶500 dilutions for 1 h. Lastly, the coverslips were incubated with DAPI (Invitrogen) at a 1∶500 dilution for 5 min. The coverslips were analyzed using a fluorescence microscope. Meanwhile, pGLUT1-GFP and pGLUT12-RFP (Fig. S4) were constructed and transfected into GMGE cells (GT1-GFP-GMGE cells and GT12-GFP-GMGE cells) respectively to detect the overexpression part of the GLUT1 and GLUT12 under the fluorescence microscope.

### The effection of the overexpression of GLUT1 or GLUT12 by RT-qPCR

Total RNA were isolated from GMGE cells which were transfected with pcDNA3.1, pcDNA3.1-GLUT1 and pcDNA3.1-GLUT12 respectively or simultaneously. RT-qPCR was performed to detect the expression of genes related to glucose or protein metabolism in GMGE cells. The sequences of the primer oligonucleotides used in this study are listed in Table. 2.

**Table 2:** Sequences of oligonucleotide primers used for RT-qPCR.

Primers names	Type	Sequences
RPS6-F	Forward	5'-CTGGGTGAAGAATGGAAGGG-3'
RPS6-R	Reverse	5'-CGAACTCTGCCATGGGTCA-3'
RPL4-F	Forward	5'-AAGATGGCGCCGAAGAAAG-3'
RPL4-R	Reverse	5'-TTTCCCGAATCAAAAATTCCA-3'
RPL22-F	Forward	5'-TCGAAGCTAGGACGTGGTGG-3'
RPL22-R	Reverse	5'-TTGGCTCCTGTGTTGTCAGC-3'
RPL35-F	Forward	5'-TTGGAAACATGTGTCGTGGG-3'
RPL35-R	Reverse	5'-GCAGATGGCGTATCGCTTCT-3'
EEF1A1-F	Forward	5'-CATCCCAGGCTGACTGTGC-3'
EEF1A1-R	Reverse	5'-TGTAAGCCAAAAGGGCATGC-3'
EEF2-F	Forward	5'-GGGCTGGTGTCTACTGGCC-3'
EEF2-R	Reverse	5'-TCAGGATTGTCCTCTGGATCG-3'
PIK3C3-F	Forward	5'-GCCAAGCATTGTTGAAGGGT-3'
PIK3C3-R	Reverse	5'-GCACCAGCCGATCTACAAAAG-3'
RHEB-F	Forward	5'-GCTAAGATGCCGCAGTCCA-3'
RHEB-R	Reverse	5'-CGTCAACGAGGATTTCCCC-3'
PRLR-F	Forward	5'-ATAGCATGGTGACCTGCATCC-3'
PRLR-R	Reverse	5'-TCTTCGGACTTGCCCTTCTC-3'
STAT5B-F	Forward	5'-GCAGCTCCAGAACACGTACG-3'
STAT5B-R	Reverse	5'-CATTGTTGGCTTCTCGGACC-3'
ß-actin-F	Forward	5'-CCAACCGTGAGAAGATGA-3'
*ß*-actin-R	Reverse	5'-CAGAGTCCATGACAATGC-3'

### Statistical Analysis

Statistical analysis was performed using the OriginLab OriginPro 9.0.0 software. Comparison of means was performed using Fisher's protected LSD test.

## Results

### Cloning of goat GLUT1 and GLUT12

Partial goat GLUT1 and GLUT12 gene sequences containing the CDS regions were amplified from goat mammary glands. The amplified GLUT1 and GLUT12 sequences and the digestion of pcDNA3.1-GLUT1 and pcDNA3.1-GLUT12 were evaluated using agarose gel electrophoresis (Fig. 1). The partial mRNA of goat GLUT1 is 2108 bp long (GenBank accession no. JQ343217), and goat GLUT12 is 1866 bp long (GenBank accession no. JQ798185). An open reading frame analysis suggested that goat GLUT1 is composed of 492 amino acids, with a molecular weight of approximately 54 kDa (Fig. S1). The DNA sequence of goat GLUT1 is 97%, 91% and 86% identical to the sequences of bovine (NM_174602.2), human (NM_006516.2) and mouse (NM_011400.3) GLUT1, respectively, and the deduced amino acid sequence of goat GLUT1 is 99%, 97% and 96% identical to the sequences of bovine (NP_777027.1), human (NP_006507.2) and mouse (NP_035530.2) GLUT1, respectively (Fig. S2). Goat GLUT12 is composed of 621 amino acids, with a molecular weight of approximately 54 kDa (Fig. S1). The partial cDNA sequence of goat GLUT12 is 97% and 84% identical to the sequences of bovine (NM_001011683.1) and human (NM_145176.2) GLUT12, respectively, and the deduced amino acid sequence of goat GLUT12 is 98% and 87% identical to the sequences of bovine (NP_001011683.1) and human (NP_660159.1) GLUT12, respectively (Fig. S2). The characteristic dileucine motif is exhibited only in the N-terminus and not in the C-terminal region (Fig. S2).

### Sequence analysis of goat GLUT1 and GLUT12

The prediction of the transmembrane helices in goat GLUT1 and GLUT12 by the TMHMM Server v. 2.0 revealed that the distribution pattern of its hydrophobic and presumed membrane-spanning segments generally favored the proposed secondary structure of GLUTs, a 12-helix model. Goat GLUT1 is predicted to present a large exoplasmic loop between TMs 6 and 7 (loop 6) and a glycosylated extracellular loop between TMs 1 and 2 (loop 1), in addition to two large exoplasmic termini. The secondary structure of the goat GLUT1 protein is similar to bovine GLUT1 (Fig. S3), and the secondary structure of the goat GLUT12 protein is similar to bovine GLUT12. Goat GLUT12 is also predicted to have two large exoplasmic loops between TMs 6 and 7 (loop 6) and between 9 and 10 (loop 9; Fig. S2). There are four potential N-glycosylation sites positioned at amino acids 375–378 (NFTS), 387–390 (NQSL), 400–403 (NLSA), and 405–408 (NDTL) of goat GLUT12 (Fig. S2). These four sites are all located in putative loop 9, which is a structural characteristic of class III members of the GLUT family. The glycosylation results are also similar for bovine GLUT12. The conserved domain analyses revealed that there are two sugar transporters and three major facilitator superfamilies (MFSs). Several other motifs, which may participate in transport activity, are also included, such as MFS transporters, cation transport proteins, and D-galactonate transporters.

### Detection of GLUT1, GLUT12, sweet1 and amino acid transporter gene expression by RT-qPCR

**Figure 2:**
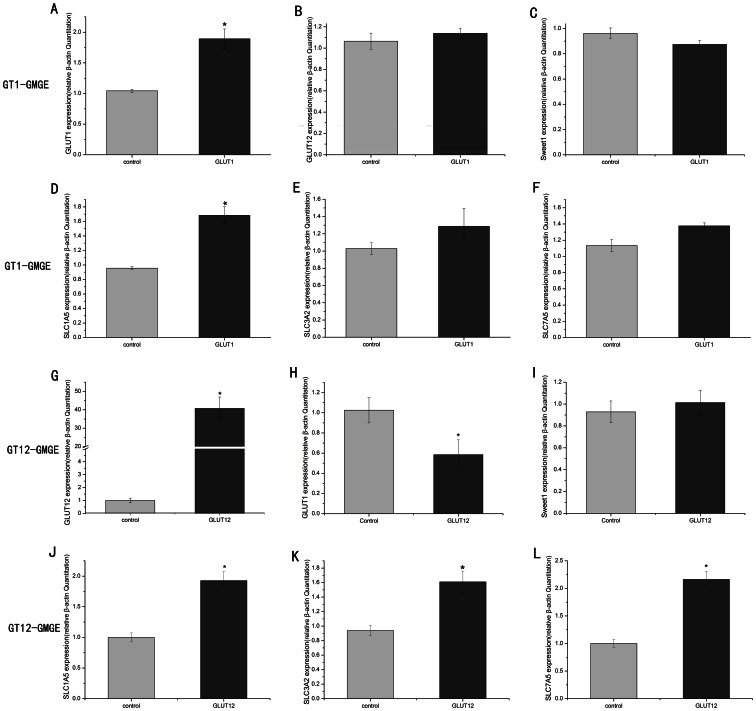
GLUT1 (A and H), GLUT12 (B and G), Sweet1 (C and I), SLC1A5 (D and J), SLC3A2 (E and K) and SLC7A5 (F and L) mRNA expression in GT1-GMGE and GT12-GMGE respectively. In GT1-GMGE, The GLUT1 (A) and SLC1A5 (B) mRNA in GT1-GMGE had significantly greater than GMGE (*P*<0.05), However the SLC7A5 (C) and SLC3A2 (D) mRNA was higher than GMGE but not significantly (*P*>0.05). In GT12-GMGE, the GLUT12 mRNA expression was increased significantly (*P*<0.01), while the GLUT1 expression was reduced significantly in GT1-GMGE (*P*<0.01). Furthermore, the SLC1A5, SLC3A2, SLC7A5 mRNA expression were all increased significantly (*P*<0.01). Data are expressed as means ± SE (n = 3). *P<0.01, compared with GMGE.

The mRNA expression of GLUT1 was increased by 80% in the GT1-GMGE cells when compared with the GMGE cells (Fig. 2), whereas no difference in GLUT12 expression was observed. Because glucose is required as a substrate for protein synthesis, we also detected the expression of three amino acid transporters: SLC1A5, SLC3A2 and SLC7A5. The mRNA of SLC7A5 and SLC3A2 in the GT1-GMGE cells increased by 20% and 25%, respectively, but was not significant when compared with the GMGE cells (Fig. 2). In the GT12-GMGE cells, the mRNA expression of GLUT12 was nearly increased by 40-fold compared with the GMGE cells, whereas GLUT1 expression was significantly decreased (*P*<0.01). Furthermore, the mRNA expression levels of the three amino acid transports were all significantly increased (*P*<0.01). Sweet1 is a recently reported sugar transporter involved in intercellular exchange and nutrition. The GMGE cells were found to express sweet1 RNA, yet this expression was not influenced by the overexpression of GLUT1 or GLUT12.

### Glucose consumption and lactose yield in GMGE, GT1-GMGE and GT12-GMGE cells

**Figure 3:**
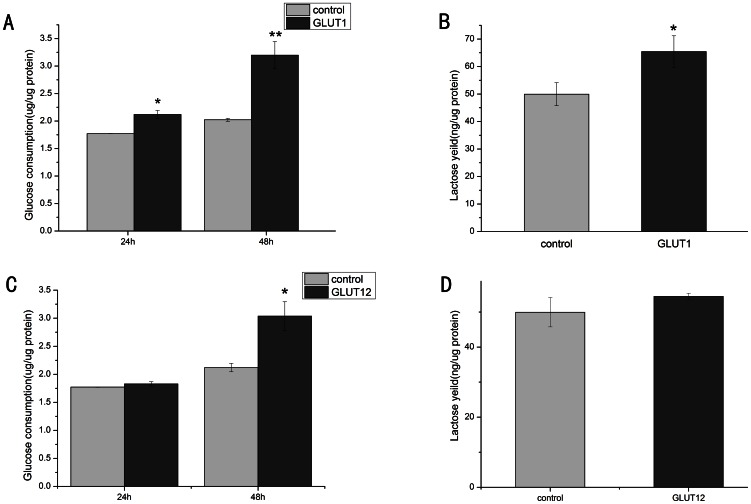
Glucose uptake (A and C) and lactose secretion (B and D) in GT1-GMGE and GT12-GMGE respectively. Glucose uptake was detected in 24 h and 48 h in GT1-GMGE and GT12-GMGE, while lactose secretion was detected in 48 h. Vertical coordinate means glucose uptake or lactose concentration and total protein radio. Horizontal coordinate means different groups. Data are expressed as means ± SE (n = 3). *P<0.01, compared with GMGE.

Glucose consumption was detected to verify the biological function of the goat GLUT1 and GLUT12 genes. Compared to the GMGE cells, the glucose consumption of the GT1-GMGE cells (2.02309±0.02674) was significantly increased in comparison to the GMGE cells (1.77139±0.00346; *P*<0.05) at 24 h and reached 3.19843±0.24797 at 48 h (*P*<0.01; Fig. 3). The mammary gland absorbs glucose to synthesize lactose, and the lactose concentration in the GT1-GMGE culture (65.44358±5.69766) was significantly higher than that in the GMGE culture (49.92578±4.19244) (*P*<0.05; Fig. 3). There was no difference in the glucose consumption between the GT12-GMGE and GMGE cells at 24 h; however, the glucose consumption increased significantly (*P*<0.01) at 48 h in the GT12-GMGE cells. The lactose concentrations between the GT1-GMGE/GT12-GMGE and GMGE culture media were not statistically significant at 48 h.

### Characterization of GLUT1 and GLUT12

**Figure 4:**
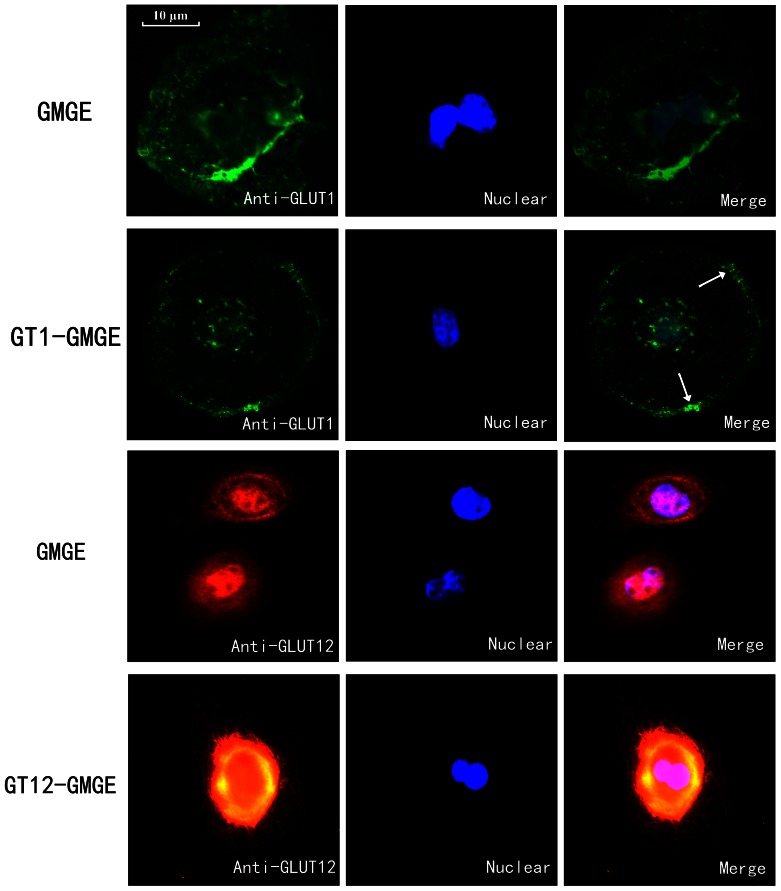
Immunofluorescence localization of goat GLUT1 and GLUT12 in GT1-GMGE and GT12-GMGE. Cells were fixed with paraformaldehyde, permeablized and incubated with antibody of GLUT1 or GLUT12. At last, the plate incubated with DAPI. The green fluorescence was scattered throughout the cytoplasm in GMGE. In GT1-GMGE cells, the total GLUT1 was mainly distributed along the plasma membrane in addition to the cytoplasm. The red fluorescence was mainly distributed around the nuclear in GMGE, while the GLUT12 expression was increased and was also mainly distributed in the perinuclear region in GT12-GMGE cells. Original magnification 400×.

The inherent part of the GLUT1, labeled with green fluorescence, was scattered throughout the cytoplasm and localized to the Golgi apparatus (Fig. 4). This result is consistent with the idea of GLUT1 being a primary transporter responsible for basal glucose uptake. In the GT1-GFP-GMGE cells, the overexpression of GLUT1 was mainly distributed around the nuclear membrane (Fig. S5). In the GT1-GMGE cells, the total GLUT1 was mainly distributed along the plasma membrane in addition to the cytoplasm and Golgi apparatus. While the inherent part of the GLUT12, labeled with red fluorescence, was scattered throughout the cytoplasm and localized around the nuclear membrane (Fig. 4). In the GT12-RFP-GMGE cells, the overexpression of GLUT12 was mainly distributed around the nuclear membrane (Fig. S5). In the GT12-GMGE cells, the total GLUT12 was also mainly distributed in the perinuclear region and cytoplasm. The distribution of goat GLUT12 was similar to bovine GLUT12, which also exhibited an intracellular localization [15].

### The genes expression in GT1-GMGE and GT12-GMGE cells

**Figure 5:**
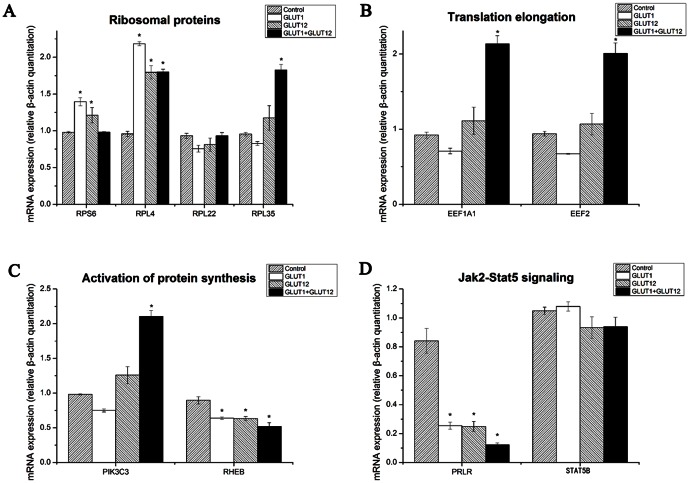
Change in expression of genes related to ribosomal proteins (A), translation elongation (B), activation of protein synthesis (C), and Jak2-Stat5 signaling (D). Data are expressed as means ± SE (n = 3). *P<0.05.

The transfection of GMGE cells could both increase the expressions of ribosomal proteins RPS6 and RPL4 significantly, which indicated that more energy will be used to synthesis ribosomal proteins and milk proteins corresponding to the excess glucose absorption (Fig. 5). Similar results were also detected for RPL35 expression in cells transfected with pcDNA3.1-GLUT1 and pcDNA3.1-GLUT12 simultaneously. However, RPL22 expression was not influenced. Eukaryotic translation elongation factor 1 alpha 1 (EEF1A1) and eukaryotic translation elongation factor 2 (EEF2) are two necessary genes for translation elongation [18]. The EEF1A1 and EEF2 expression were both up-regulated significantly in GMGE cells transfected with pcDNA3.1-GLUT1 and pcDNA3.1-GLUT12 simultaneously. However, transfection with pcDNA3.1-GLUT1 and pcDNA3.1-GLUT12 respectively did not affect the EEF1A1 and EEF2 expression in GMGE cells.

The phosphoinositide-3-kinase class 3 (PIK3C3) expression was significantly up-regulated to activate protein synthesis in GMGE cells transfected with pcDNA3.1-GLUT1 and pcDNA3.1-GLUT12 simultaneously. However, Ras homolog enriched in brain (RHEB) expression was significantly down-regulated. Furthermore, the STAT5B expression was not changed, while PRLR expression was significantly decreased in GMGE cells transfected with pcDNA3.1-GLUT1 and pcDNA3.1-GLUT12 respectively or simultaneously.

## Discussion

GLUTs are expressed in every cell of the body and provide the metabolic energy and building blocks for the synthesis of biomolecules and control glucose utilization, glucose production and glucose sensing [19]. GLUT1, responsible for basal glucose uptake, is considered to be the primary monosaccharide transporter. In contrast, GLUT12 is mainly expressed in skeletal muscle, adipose tissue, the small intestine and placenta [14]. Rogers *et al.* speculated that human GLUT12 is expressed in prostate cancer and breast cancer [20], whereas it is absent in normal prostate and expressed at very low levels in normal breast tissue [21]. However, the biological function of GLUT12 is not clear. Moreover, no data regarding goat GLUTs are currently available. In this study, we cloned goat GLUT1 and GLUT12 from goat mammary gland tissue. The prediction of the transmembrane helices demonstrated that both goat GLUT1 and GLUT12 have 12 transmembrane structures and belong to the class I and III proteins of the GLUT family, respectively. Goat GLUT1 and GLUT12 are highly homologous to other mammalian GLUTs and exhibited all of the motifs that are presumably required for sugar transport activity [22]–[24].

We inserted goat GLUT1 and GLUT12 into the pcDNA3.1 (+) plasmid and transfected these constructs into GMGE cells to assess the functions of goat GLUT1 and GLUT12 in mammary gland cells. In the GT1-GMGE cells, the mRNA expression of GLUT1 was significantly increased, whereas the expression of GLUT12 mRNA was unchanged. In the GT12-GMGE cells, the GLUT12 expression increased significantly, and the GLUT1 expression decreased significantly. These results demonstrated that the transcription of goat GLUT1 and GLUT12 was driven by the cytomegalovirus (CMV) promoter. Because GLUT12 expression is restricted mainly to insulin-sensitive tissues, it is postulated to be a second insulin-responsive glucose transporter, along with GLUT4 [14]. The GLUT4 protein also acts in a cooperative manner with GLUT1, which is evident in insulin-sensitive tissues (fat and muscle) where the GLUT1 protein is localized to the plasma membrane and the tissue-specific GLUT 4 is distributed in an intracellular compartment in the basal state [25]. Furthermore, GLUT1 may also influence and cooperate with GLUT12. In this study, GLUT12 expression influenced the transcription of GLUT1, which was responsible for the basal glucose uptake of the cells. This decreased GLUT1 expression and increased GLUT12 expression in the GT12-GMGE cells implies that GLUT12 may provide a back-up system for GLUT1, inducing an increase in glucose consumption at 48 h. Given that GLUT12 is distributed in the cytoplasm and GLUT1 is responsible for the absorption of glucose from the basal plasma membrane of epithelium, this may be related to lactose synthesis.

Solute carrier family 1 member 5 (SLC1A5) encodes ASCT2, the major amino acid transporter, which transports the neutral amino acids Ala, Gly, Leu, Ile, Val, Ser, Cys, Thr and Pro. The mRNA expression of SLC1A5 in the GT1-GMGE and GT12-GMGE cells was significantly increased compared to the GMGE cells, indicating that protein synthesis and metabolism in the GT1-GMGE and GT12-GMGE cells were higher than in the GMGE cells, processes that are related to increased glucose uptake [26]. We also found that the mRNA levels of SLC7A5 and SLC3A2 were also increased. Solute carrier family 7 member 5 (SLC7A5) codes for LAT1, which transports cationic amino acids; solute carrier family 3 member 2 (SLC3A2) codes for 4F2hc, which is an activator of dibasic and neutral amino acid transporters. Two AATs form the functional heterodimer AA transporter and have a reciprocal regulatory connection with mTOR in the regulation of protein synthesis and cell viability, most likely through the active transport of Leu [27]. mTOR controls ribosome biogenesis and cell growth in regulatory pathways. New research shows that the mTOR pathway is influenced by the intracellular concentration of ATP, independent of the abundance of amino acids, and that mTOR itself is an ATP sensor [26]. Thus, the additional glucose due to the overexpression of GLUT1 may promote protein synthesis and strengthen the cell viability. 4F2hc is a membrane protein that interacts with GLUT1 and is likely to be involved in GLUT1 stabilization and contributes to the regulation of glucose metabolism [28]. The results of our study corroborate the findings of Ohno *et al.* who reported GLUT1 functions require the assistance of 4F2hc [28].

Many proteins are targeted to the plasma membrane, including human GLUT4 and GLUT12, and are transported from the endoplasmic reticulum through the Golgi apparatus to the cell surface [4], [29]. Human GLUT4 and GLUT12 contain several potential targeting motifs that control their localization. Dileucine motifs are present in both the NH_2_ and COOH termini in human GLUT12. In the absence of insulin, GLUT12 is localized to a perinuclear site in MCF-7 cells [14] and to the Golgi network in stably GLUT12-expressing HEK293 cells [30]. Conversely, human GLUT12 is translocated to the plasma membrane in response to insulin in skeletal muscle cells [31]. However, a dileucine motif is only exhibited in the N-termini of bovine and goat at positions similar to the GLUT4 FQQI motifs. Moreover, a severe resistance to the stimulatory action of insulin on glucose metabolism was noted in ruminant animals compared to rats [32]. The significance of the absence of the dileucine motif in the C-terminal region and the low sensitivity to insulin stimulation of ruminant animals may be related to the cytoplasmic distribution of goat GLUT12. Similar results were also observed for bovine GLUT12 [15].

Our immunofluorescence studies show that GLUT1 is localized to the cell membrane and around the nucleus and that the brightness around the nucleus was significantly higher than in other parts of the cell, results that are consistent with other research [4], [28], [33]. As mammary epithelial cells undergo vigorous protein synthesis, they contain a large number of Golgi apparati around the nucleus. The localization of GLUT1 to the membrane of epithelial cells was different between the GT1-GMGE and GMGE cells. Furthermore, the overexpression of GLUT1 targeted the protein to the plasma membrane more than in the control GMGE cells, explaining the observed increase in glucose uptake by the GT1-GMGE cells in this study.

GLUT1 provides cells with glucose, but its abnormal expression also has a relationship with tumor growth and diabetes [9], [10], [34], [35]. We have shown that hypoxia and low sugar can induce GLUT1 expression and that the growth and metabolism of cancer cells expressing GLUT1 is rather higher than in other cells. Recently, further research indicates that GLUT has both glucose transporter functions and plays an important role in immunity [36]–[39]. GLUT1 is a receptor of HTLV infection in CD4+T cells [37], and HIV-1 infection in CD4+T cells is abrogated in the absence of surface GLUT1 [40]. Recent studies [7], [22], [41], [42] have found that GLUT1 is most abundantly expressed in mammary glands and that the levels increase during pregnancy and peak during lactation, results that may be related to the abundant synthesis of proteins and lactose in breast tissue. In this study, using breast epithelial cells, the data for glucose uptake in the GT1-GMGE cells confirmed that the cloned GLUT1 possesses glucose transport activity, a result that corresponded to the high expression of GLUT1 mRNA in the GT1-GMGE cells. Glucose is required to provide substrate and energy for the synthesis of milk lactose. Therefore, the lactose yield could indirectly reflect GLUT1 function in mammary epithelial cells. In our study, the lactose content in the medium of the GT1-GMGE cells also increased, which may explain the additional glucose in the cells.

The overexpression of GLUTs could influence some genes’ expressions which were related to the glucose and protein metabolism in GMGE cells. This observation might suggest some ribosomal components related to milk protein synthesis could be regulated by glucose absorption. The overexpression of EEF1A1 and EEF2 was only detected in GMGE cells transfected with pcDNA3.1-GLUT1 and pcDNA3.1-GLUT12 simultaneously, which may be attributed to that GLUT1 was mainly distributed in cell membrane, while GLUT12 was always expressed in endoplasmic reticulum or Golgi apparatus. Enough glucose in endoplasmic reticulum or Golgi apparatus alone was unable to guarantee the activation of protein synthesis. Only when glucose is both abundant in the cytoplasm and Golgi apparatus, the translation elongation will be promoted to start protein synthesis. Vps34, encoded by PIK3C3, is necessary for mTORC1 activity in response to amino acids sensing in cultured cells [18]. The overexpression of GLUTs could enhance AATs expression to absorb more amino acids, which would then induce the up-regulation of PIK3C3. As inhibitor of mTOR signaling [18], the decrease expression of RHEB could in turn stimulate the activation of protein synthesis. Besides mTOR signaling, the Jak-Stat signaling pathway could also take part in glucose and protein metabolism in MGE cells [43]. However, the overexpression of GLUT1 or GLUT12 did not affect STAT5B expression. Moreover, PRLR expression was significantly down-regulated compared to control group. We guess that enhanced AATs expression stimulated the absorption of amino acids, and the GMGE cells used the PRLR pathway as a negative regulation way to balance the intracellular condition. The detailed mechanism should be determined in future.

## Supporting Information

Figure S1 The partial cDNA and deduced amino acid sequence of GLUT1 and GLUT12. In the cDNA sequence uppercase letters represent the 5' and 3' untranslated regions and lowercase letters represent the coding region. The predicted amino acid sequences shown in uppercase letters are beneath the coding sequences. (A) N-45 was the predicted N-glycosylation site highlighted in the red circle. (B) N-375, 387, 400 and 405 were the predicted N-glycosylation sites highlighted in the red circle.
(TIF)

Figure S2 Multiple sequence alignment of the deduced amino acid sequences of goat GLUT1 and GLUT12 with other species. The alignments were performed with the CLUSTALW 2.1. An * (asterisk) indicates positions which have a single, fully conserved residue. A : (colon) indicates conservation between groups of strongly similar properties-scoring >0.5 in the Gonnet PAM 250 matrix. A. (period) indicates conservation between groups of weakly similar properties-scoring  = <0.5 in the Gonnet PAM 250 matrix.
(TIF)

Figure S3 Prediction of transmembrane helices analysis of goat GLUT1 (A), and bovine GLUT1 (B), goat GLUT12 (C) and bovine GLUT12 (D). Red line means transmembrane region, blue line means inside region and pink line means outside region. Vertical coordinate means probability of transmembrane helices, and horizontal coordinate means AA sequence.
(TIF)

Figure S4 pGLUT1-GFP (A) and pGLUT12-RFP (B) vector construction diagrams.
(TIF)

Figure S5 Detection of GLUT1 (A: inherent GLUT1, B: the overexpression of GLUT1, C: the total expression of GLUT1) and GlUT12 in GMGE cells (D: inherent GLUT12, E: the overexpression of GLUT12, F: the total expression of GLUT12). pGLUT1-GFP and pGLUT12-RFP were transfected into GMGE cells (GT1-GFP-GMGE cells and GT12-GFP-GMGE cells) respectively to detect the overexpression part of the GLUT1 (B) and GLUT12 (E) under the fluorescence microscope. The overexpression of GLUT1 and GLUT12 were both mainly distributed around the nuclear membrane.
(TIF)
