# Supplementary figures and images for: Functional Analyse of GLUT1 and GLUT12 in Glucose Uptake in Goat Mammary Gland Epithelial Cells
Source: PLoS One. 2013 May 28;8(5):e65013. doi: 10.1371/journal.pone.0065013 (PMC3665807; doi:10.1371/journal.pone.0065013)

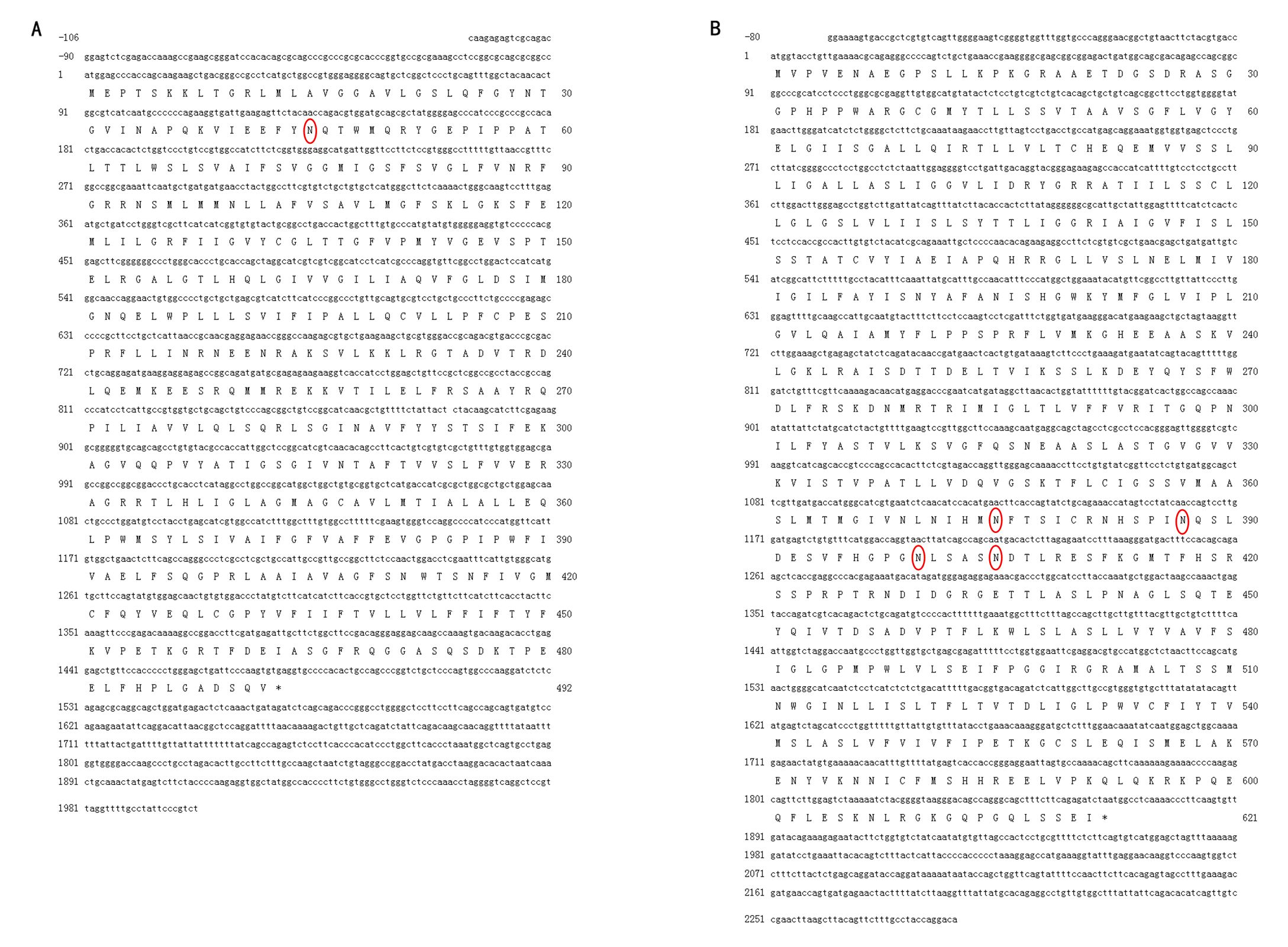

Supplement: Figure S1 — The partial cDNA and deduced amino acid sequence of GLUT1 and GLUT12. In the cDNA sequence uppercase letters represent the 5' and 3' untranslated regions and lowercase letters represent the coding region. The predicted amino acid sequences shown in uppercase letters are beneath the coding sequences. (A) N-45 was the predicted N-glycosylation site highlighted in the red circle. (B) N-375, 387, 400 and 405 were the predicted N-glycosylation sites highlighted in the red circle. (TIF) [file pone.0065013.s001.tif]

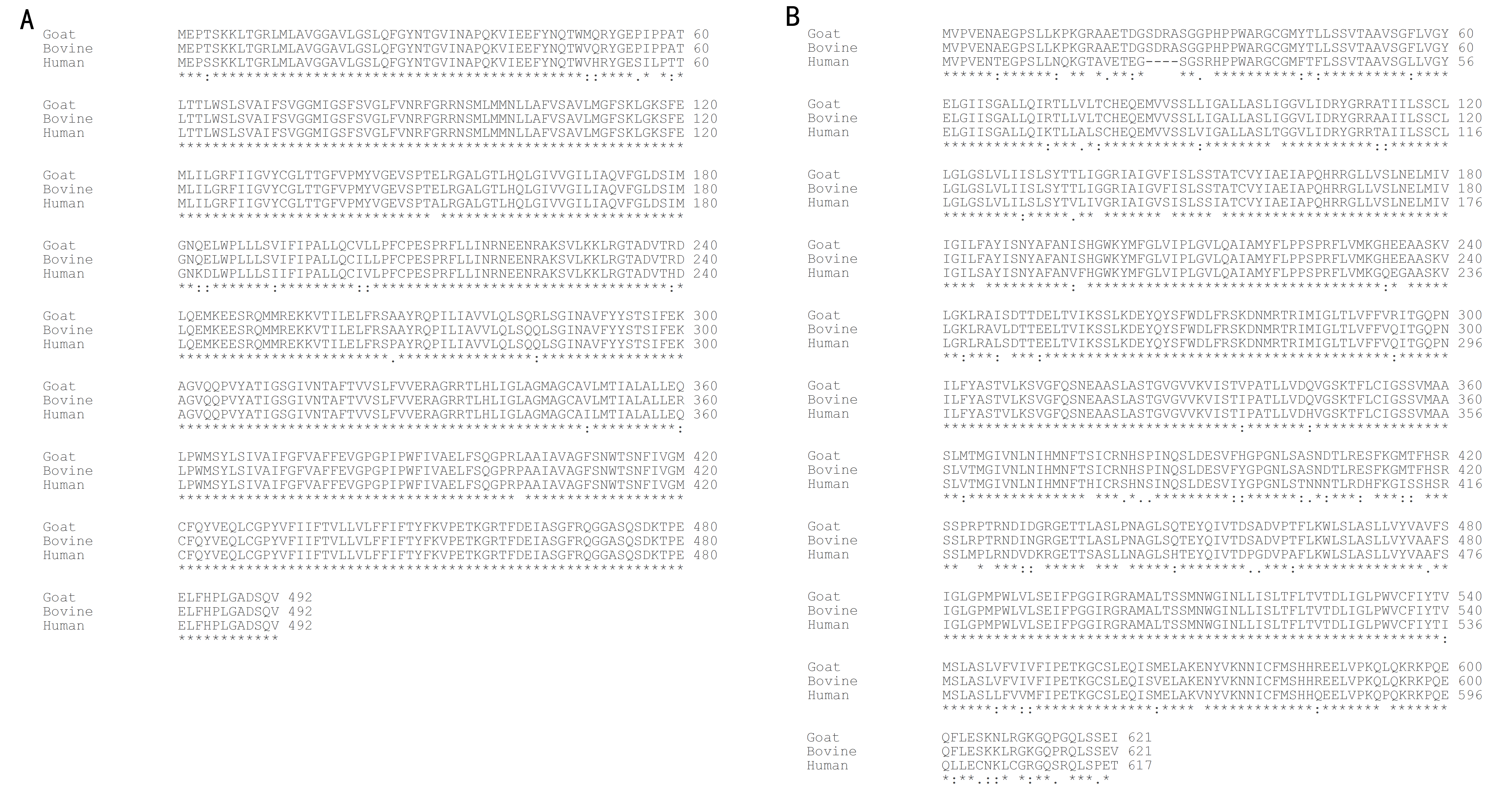

Supplement: Figure S2 — Multiple sequence alignment of the deduced amino acid sequences of goat GLUT1 and GLUT12 with other species. The alignments were performed with the CLUSTALW 2.1. An * (asterisk) indicates positions which have a single, fully conserved residue. A : (colon) indicates conservation between groups of strongly similar properties-scoring >0.5 in the Gonnet PAM 250 matrix. A. (period) indicates conservation between groups of weakly similar properties-scoring = <0.5 in the Gonnet PAM 250 matrix. (TIF) [file pone.0065013.s002.tif]

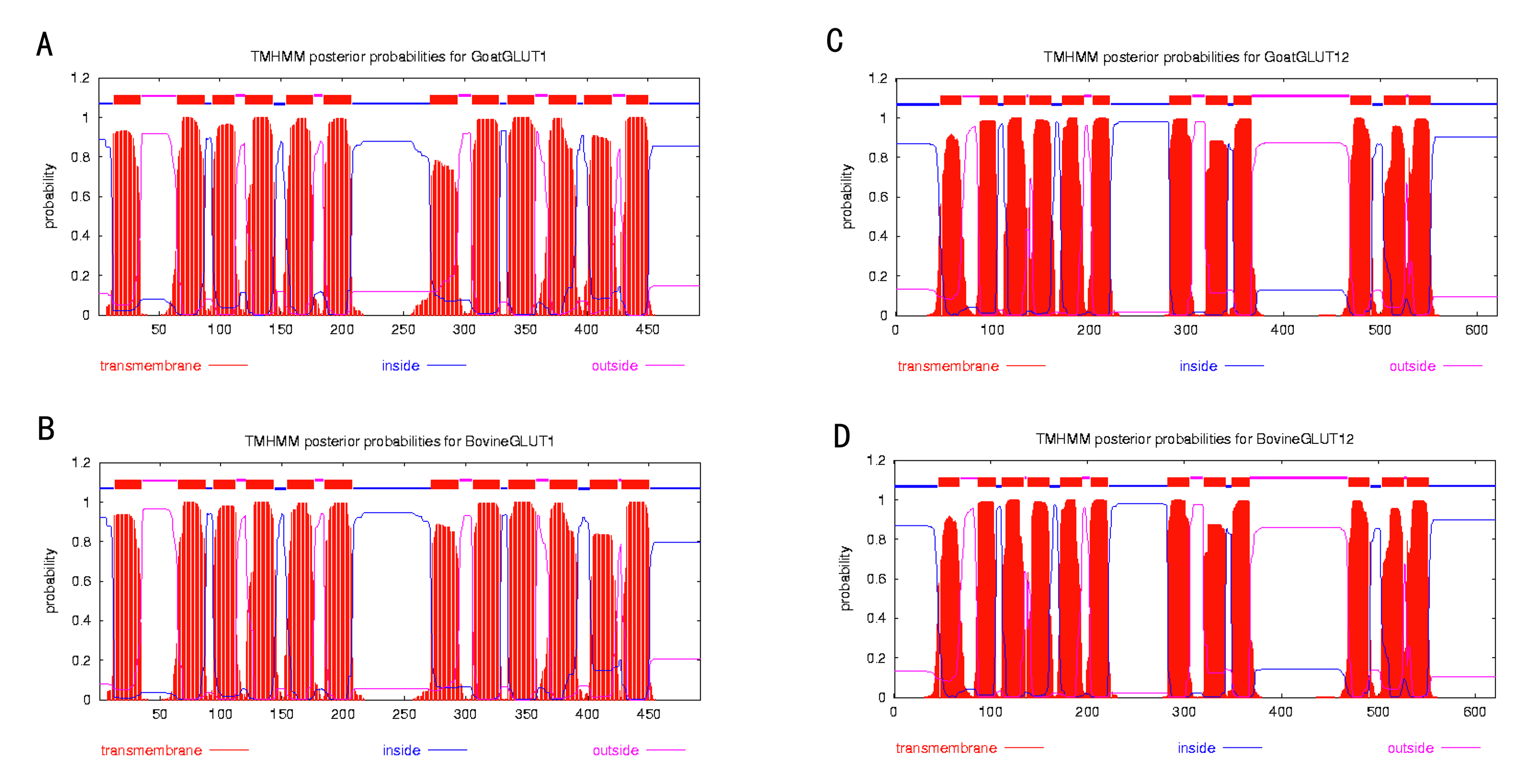

Supplement: Figure S3 — Prediction of transmembrane helices analysis of goat GLUT1 (A), and bovine GLUT1 (B), goat GLUT12 (C) and bovine GLUT12 (D). Red line means transmembrane region, blue line means inside region and pink line means outside region. Vertical coordinate means probability of transmembrane helices, and horizontal coordinate means AA sequence. (TIF) [file pone.0065013.s003.tif]

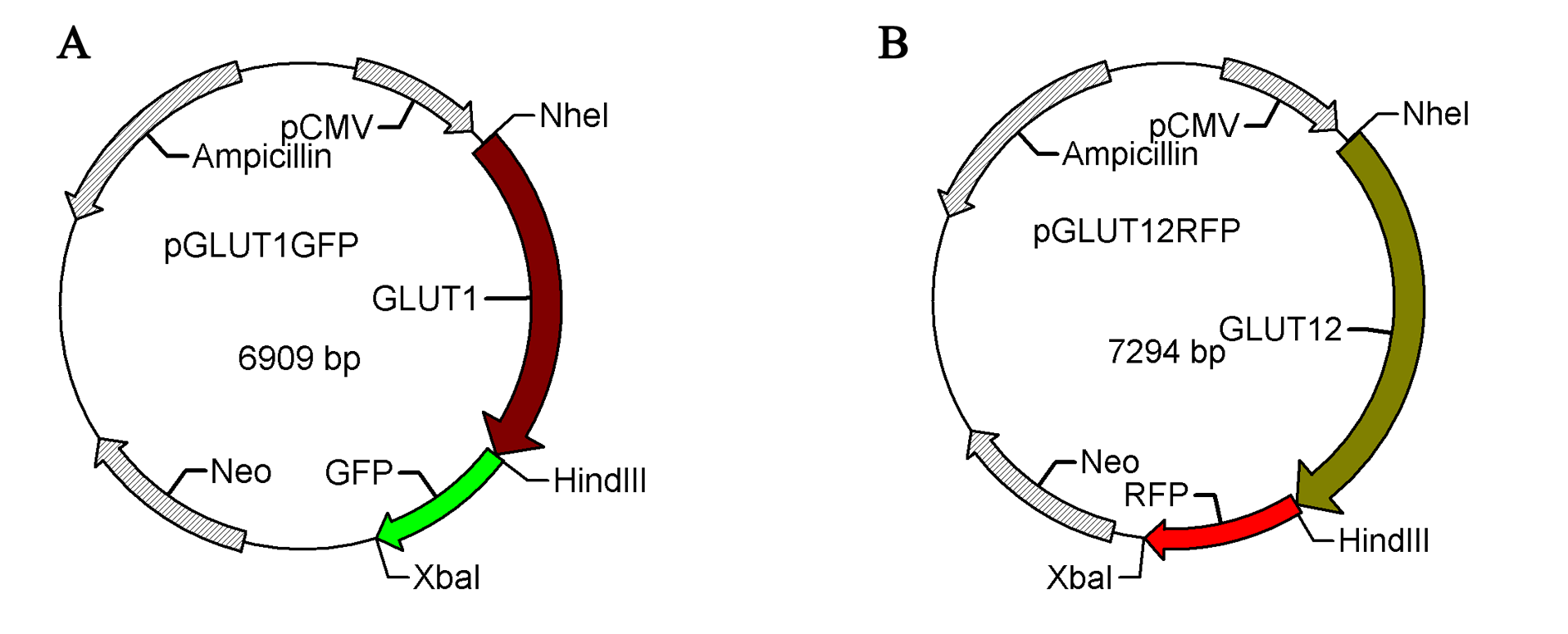

Supplement: Figure S4 — pGLUT1-GFP (A) and pGLUT12-RFP (B) vector construction diagrams. (TIF) [file pone.0065013.s004.tif]

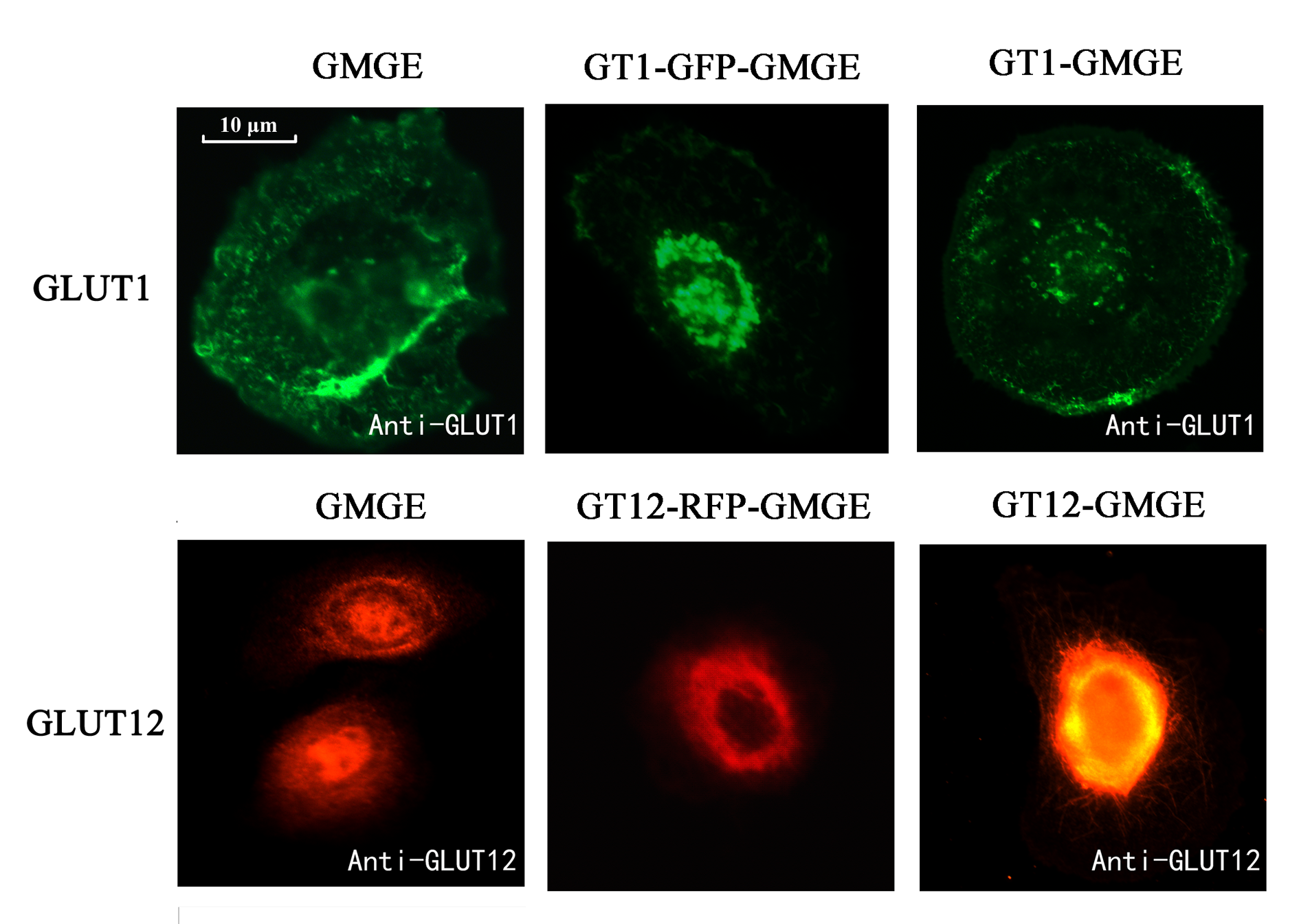

Supplement: Figure S5 — Detection of GLUT1 (A: inherent GLUT1, B: the overexpression of GLUT1, C: the total expression of GLUT1) and GlUT12 in GMGE cells (D: inherent GLUT12, E: the overexpression of GLUT12, F: the total expression of GLUT12). pGLUT1-GFP and pGLUT12-RFP were transfected into GMGE cells (GT1-GFP-GMGE cells and GT12-GFP-GMGE cells) respectively to detect the overexpression part of the GLUT1 (B) and GLUT12 (E) under the fluorescence microscope. The overexpression of GLUT1 and GLUT12 were both mainly distributed around the nuclear membrane. (TIF) [file pone.0065013.s005.tif]
